# Initial Implementation of the My Heart, My Life Program by the National Heart Foundation of Australia: Pilot Mixed Methods Evaluation Study

**DOI:** 10.2196/43889

**Published:** 2023-10-05

**Authors:** Samia Kazi, Chloe Truesdale, Pauline Ryan, Glen Wiesner, Garry Jennings, Clara Chow

**Affiliations:** 1 The University of Sydney Sydney Australia; 2 Westmead Applied Research Centre Westmead Australia; 3 The National Heart Foundation Melbourne Australia

**Keywords:** cardiology, prevention, digital health, heart, text message, text messaging, SMS, health communication, demographic, preventative, cardio

## Abstract

**Background:**

Coronary heart disease (CHD) remains the leading cause of death in Australia, with a high residual risk of repeat events in survivors. Secondary prevention therapy is crucial for reducing the risk of both death and other major adverse cardiac events. The National Heart Foundation of Australia has developed a consumer-facing support program called My Heart, My Life (MHML) to address the gap in the secondary prevention of CHD in Australia. The MHML pilot program supplies advice and support for both patients and their caregivers, and it was conducted over 8 months from November 2019 to June 2020.

**Objective:**

This study aims to describe and examine the implementation of a novel multimodality secondary CHD prevention pilot program called MHML, which was delivered through booklets, text messages, emails, and telephone calls.

**Methods:**

This pilot study consists of a mixed methods evaluation involving surveys of participants (patients and caregivers) and health professionals, in-depth interviews, and digital communication (SMS text message, electronic direct mail, and call record analytics). This study was performed in people older than 18 years with acute coronary syndrome or angina and their caregivers in 38 Australian hospitals from November 2019 to June 2020 through the National Heart Foundation of Australia web page. The main outcome measures were reach, accessibility, feasibility, barriers, and enablers to implementation of this program.

**Results:**

Of the 1004 participants (838 patients and 164 caregivers; 2 missing), 60.9% (608/1001) were males, 50.7% (491/967) were aged between 45 and 64 years, 27.4% (276/1004) were from disadvantaged areas, 2.5% (24/946) were from Aboriginal or Torres Strait Islander background, and 16.9% (170/1004) reported English as their second language. The participants (patients and their caregivers) and health professionals reported high satisfaction with the MHML program (55/62, 88.7% and 33/38, 87%, respectively). Of the 62 participants who took the survey, 88% (55/62) used the text messaging service and reported a very high level of satisfaction. Approximately 94% (58/62) and 89% (55/62) of the participants were satisfied with the quick guide booklets 1 and 2, respectively; 79% (49/62) were satisfied with the monthly email journey and 71% (44/62) were satisfied with the helpline calls. Most participants reported that the MHML program improved preventive behaviors, that is, 73% (45/62) of them reported that they maintained increased physical activity and 84% (52/62) reported that they maintained a healthy diet even after the MHML program.

**Conclusions:**

The findings of our pilot study suggest that a multimodal support program, including digital, print, phone, and web-based media, for the secondary prevention of CHD is useful and could be a potential means of providing customized at-scale secondary prevention support for survivors of acute coronary syndrome.

## Introduction

Coronary heart disease (CHD) causes the greatest burden of disease and remains the leading cause of death in Australia [1]. The risk of a repeat major adverse cardiac event (MACE) in survivors of acute coronary syndrome (ACS), which includes a repeat myocardial infarction or stroke, is more than 20% in a 2-year period [2]. Secondary prevention therapies in the form of medications and cardiac rehabilitation are crucial for reducing the risk of both death and MACE [3]. Unfortunately, research from Australia demonstrates a decline in the use of secondary prevention medication therapy from as soon as 6 months after discharge [3]. Many patients are discharged without advice on secondary prevention and do not adhere to medications; moreover, only 1 in 3 eligible patients attend cardiac rehabilitation [4]. Developing innovative methods to improve this situation is urgently required to reduce the burden of this disease.

The benefits of secondary prevention programs for CHD, which include counselling, education, and exercise components, have been proven to reduce the risk of repeat MACE [5]. Aligned with this, the National Heart Foundation of Australia (NHFA) has developed a consumer-facing support program called as My Heart, My Life (MHML) to address the gap in the secondary prevention of CHD in Australia. The MHML pilot program supplies advice and support for both patients and their caregivers and was conducted over 8 months from November 2019 to June 2020.

The MHML program was initiated to complement existing support programs such as cardiac rehabilitation and was designed to improve patients’ access to health information and support Australians during and after their cardiac events to facilitate safer transitions of care and enhanced access to secondary prevention support [3]. This program is expected to contribute to improvements in multiple outcomes over time. These include increasing the confidence and knowledge of self-management of CHD by patients, improving health behavior and quality of life of people with CHD, and raising awareness of the NHFA resources. This paper outlines the implementation of the pilot program and the barriers and enablers, with the aim to assess feasibility and guide improvements to successfully expand the program.

The specific objectives of this study were to (1) describe the uptake of the MHML program, including reach and accessibility, (2) describe the characteristics of participants accessing the MHML program, (3) examine and report the implementation of the MHML pilot program with regard to feasibility, (4) provide insight into the acceptability of the patient support journey, and (5) provide information on the barriers and enablers to wide-scale implementation of this program.

## Methods

### Ethics Approval

This study received ethics approval from the University of Sydney Human Research Ethics Committee (approval 2021/771) for the project title “My Heart, My Life Support Program Pilot Evaluation.”

### Study Design

This study had a mixed methods design involving quantitative and qualitative data collection. The term participants refers to patients and their caregivers in this paper.

### MHML Program

The MHML is a multimodal program, merging existing and new resources, delivered at no cost to participants, as outlined in Table 1 and Multimedia Appendix 1. The goal of the MHML program is to support and provide information at critical points in the patient journey with different modalities to allow individualization of exposure to key messages. The NHFA also aims to overcome barriers to secondary prevention, including geography, cardiac rehabilitation access, digital technology, and health literacy. Onboarding was initially intended to be facilitated by hospital staff caring for patients with ACS; however; for a short period, self-enrollment was allowed via the NHFA website.

Print resources were provided through 2 booklets. Part 1: “Your Quick Guide to Heart Attack and Angina,” which was given in the hospital, was designed to provide individualized information upon discharge. The booklet contained a record-keeping tool for documentation of risk factors, nearest cardiac rehabilitation program, driving restrictions, and individual goals. Part 2 “Living Well With Heart Disease: Heart Attack and Angina” was mailed to the enrolled participants only. This booklet contained detailed information on long-term risk factor management, nutrition, physical activity, psychosocial well-being, and returning to pre-event quality of life.

The digital resources consisted of 2 components. The first component was a mobile phone text messaging program based on the Heart Foundation grant-in-aid–supported TEXT ME (tobacco exercise and diet messages) [6] and delivered via the TextCare platform [7]. This text messaging program comprised 4 heart health text messages per week for 6 months with message content customized to a participant’s risk factors. Messages included prompts and tips to encourage heart-healthy behaviors and habits and web links to further information (eg, diet, exercise, blood pressure/cholesterol, other risk factors including smoking as relevant). The second component was an email journey comprising 8 customized emails sent over 6 months, which included links to key heart health information, advice, narratives, recipes, and support services.

**Table 1:** Summary of the resources used for My Heart, My Life program.

Resource	Description
First patient educational booklet: part 1	A 50-page booklet called “Your Quick Guide to Heart Attack and Angina” was provided to patients by the health professionals in hospitals or health services, which covered individual details, including hospital team, information on coronary heart disease, risk factors, cardiac rehabilitation, medications, warning signs of angina and myocardial infarction, follow-up plans, and a heart dictionary. Examples of excerpts from the guide are given in Multimedia Appendix 1.
Second patient booklet: part 2	A 48-page booklet called “Living Well With Heart Disease: Heart Attack and Angina” was delivered by mail to the patient’s home. This booklet provides help with managing clinical and lifestyle risk factors, emotional and social well-being, and resuming everyday activities after a cardiac event or diagnosis.
A health information–based text message service	Participants received 4 heart health text messages per week for 6 months in their mobile phones. The SMS text message content prompted heart-healthy behaviors and habits and included links to other important health information. It was based on the TEXT ME (tobacco exercise and diet messages) [6] program and delivered via the TextCare platform (University of Sydney). The intervention provided semipersonalized text messages with advice, motivation, and information related to healthy eating patterns, increased physical activity, and if applicable, encouraging smoking cessation. Examples of SMS text messages sent to participants are given in Multimedia Appendix 1.
Helpline outbound support calls	Two helpline outbound calls were provided by trained health professionals, with the first call being within 2-4 weeks of enrollment. The calls explored the patient/caregiver health admission, provided support and reassurance, delivered information, including modifying risk factors, discussed questions relating to posthospital care, and assisted with navigating the health system. The second phone call offered at 3 months was to check on recovery and answer any outstanding information needs.
Email support	An email journey commenced soon after patients or caregivers enrolled in the program, which included 8 bespoke emails delivered over a 6-month period. Three emails were delivered in the first month, and then monthly emails were delivered. The emails provided links to key heart health information and advice, narratives, recipes, and support services.

The enrolled participants also received 2 calls by trained health professionals from the NHFA helpline. The calls provided individualized support and reassurance based on the NHFA’s “six steps to cardiac recovery” resource. The first call, within 2-4 weeks of enrollment, discussed the hospital admission, rehabilitation options, information on risk factors, and navigating the health system. The second phone call at 3 months was to address outstanding information needs. If the first call was unanswered, an SMS text message was sent to allow the participant to return the call at their convenience. The COVID-19 pandemic decreased face-to-face interactions in the hospital and reallocation of resources at NHFA. This reduced the opportunity for promotion and engagement with hospitals and therefore patient and caregiver recruitment and initial delay by 2 weeks in meeting helpline call-timing goals.

### Setting and Participants

In this pilot program, 38 Australian hospitals (Multimedia Appendix 2) from diverse locations participated. The NHFA supported the hospitals by delivering local education sessions regarding information for the program and provided health professional conversation guides to prioritize key messages after a CHD admission. The inclusion criteria for the program included adults and their caregivers older than 18 years presenting to the hospital with a non–ST-segment elevation myocardial infarction, ST-segment elevation myocardial infarction, or angina within the last 12 months. Caregivers were family or friends involved in the cardiac journey of the participants and could also be enrolled. Health professional staff in hospital recruited these participants during their admission by making them aware of the MHML program, and once aware, participants and their caregivers could choose to (1) send a text message to a designated mobile number, which automatically provided a link to a web-based enrollment form; (2) phone the helpline to verbally complete the enrollment form; and (3) access the web-based form directly with a link. Other participants were alerted to the pilot program via a pop-up prompt on targeted pages of the NHFA website and were provided with the web-based form link for 3 months of the recruitment phase.

### Data Collection

Data were collected by the NHFA insights team, which was separate from the MHML implementation team. Participant data were collected at baseline, at 3 months, and toward the end of the pilot study across an 8-month evaluation period. Not all participants completed the 3-month survey, as the evaluation occurred 8 months into onboarding. Surveys comprised questions on acceptability, usefulness, knowledge, behavior change, and usage of intervention components (Table S1 of Multimedia Appendix 3). The interviews explored if the program enhanced participant knowledge and provided support to positively influence their recovery and ongoing management and collected data on barriers and enablers to implementation. Health professionals, including cardiac nurses and doctors, were interviewed at 4-6 months to assess the accessibility, improvements, degree of integration into practice, and feedback on program content (Table S2 of Multimedia Appendix 3).

### Data Analysis

Quantitative data analysis used descriptive statistics. Chi-square goodness-of-fit tests were used to compare the MHML sample with the general population to assess representativeness for various demographic variables (significance set at *P*<.05). Analytics reports from the TextCare text messaging platform, email journey, and helpline were analyzed, and the feedback log was analyzed according to themes. Thematic analysis was performed to survey open-ended questions and interviews. A results matrix was used to triangulate the different data sources and methods by each of the key evaluation questions. This matrix was used to assess where there were similarities or any differences by data source and by method and enabled the development of overarching key findings.

## Results

### Participant Demographics

**Figure 1:**
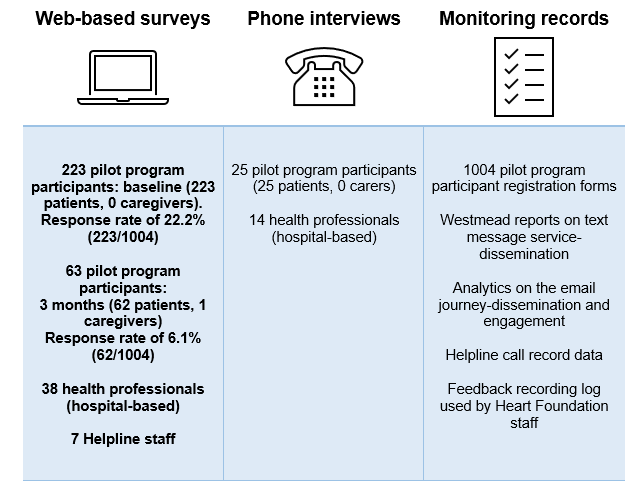
Evaluation of the participants and data sources. Response rate was calculated based on those who had been involved in the pilot program for 3 months or more at the end of the pilot period.

A total of 1004 participants (2 missing data), consisting of patients (838/1002, 83.6%) and caregivers (164/1002, 16.3%), were enrolled in this pilot study (Figure 1). Most participants were males (608/1001, 60.9%) and were aged 45-64 years (491/967, 50.7%). Most of them (763/1004, 76%) had recent (in the last 12 months) ACS (Table 2).

**Figure 2:**
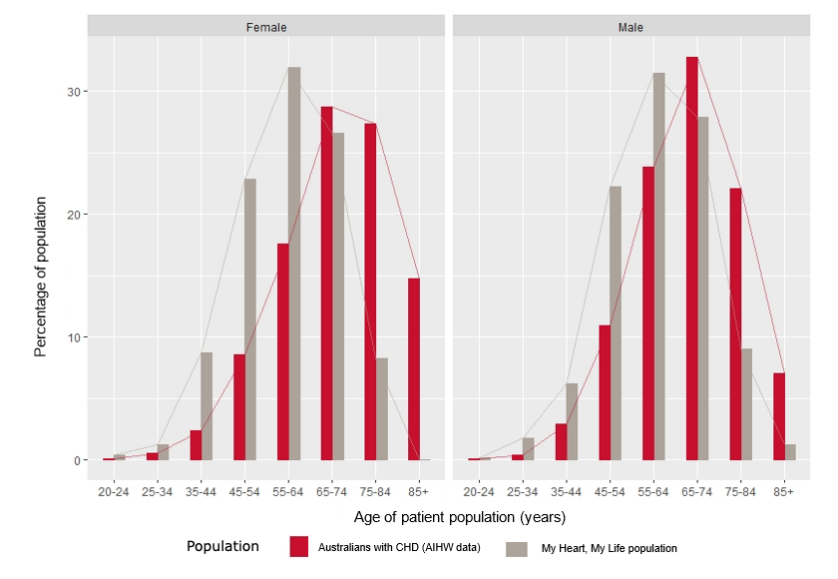
Age and sex distribution of pilot program participants relative to the national hospitalization rates for coronary heart disease. AIHW: Australian Institute of Health and Welfare; CHD: coronary heart disease.

**Figure 3:**
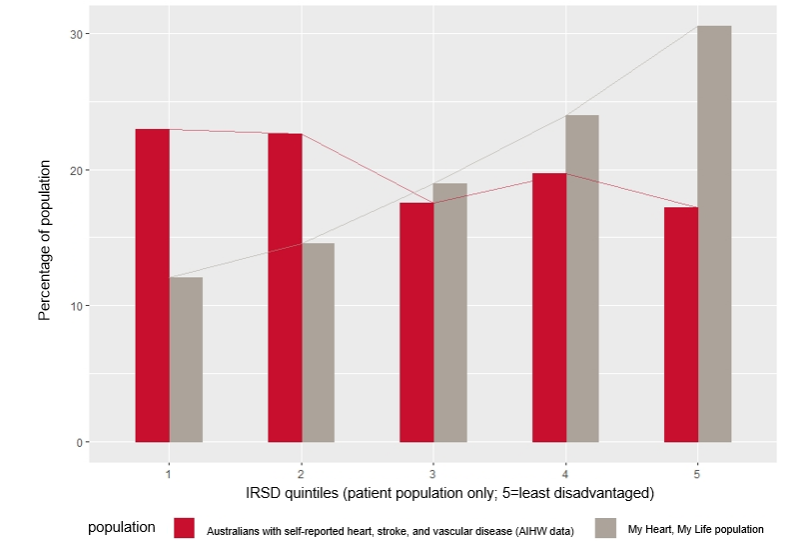
Distribution of the pilot program participants based on the Index of Relative Socioeconomic Disadvantage Quintiles relative to the national population of people with cardiovascular disease [8]. AIHW: Australian Institute of Health and Welfare; IRSD: Index of Relative Socioeconomic Disadvantage.

Of the total patient population, 69.8% (584/836) were males (2 preferred not to answer), while 85.9% (140/164, 1 missing) of the caregivers who enrolled were females. The proportion of the male patients in this program is representative of the male hospitalization data, which is higher for CHD, and suggested that the program was well accessed by both men and women. Overall, 27.6% (276/1004) of the patients lived in disadvantaged areas, 2.5% (24/946) were Aboriginal or Torres Strait Islander people, and 16.9% (170/1004) reported English as their second language. The ratio of men to women in this study was similar to that of the national heart disease hospitalized population [1,8]. The MHML sample population appeared younger (*P*<0.001) (Figure 2) and less disadvantaged (*P*<0.001) than the overall population with CHD in Australia (Figure 3).

**Table 2:** Demographics of the pilot study population (N=1004).

			Values, n (%)
Males (n=1001)			608 (60.7)
**Age group (years) (n=967)**			
	<45	139 (14.4)	
	45-64	491 (50.7)	
	65+	337 (34.9)	
Aboriginal or Torres Strait Islander (n=946)			24 (2.5)
English as a second language^a^ (N=1004)			170 (16.9)
Myocardial infarction (n=1001)			765 (76.4)
Angina (n=1001)			236 (23.5)
Patients (n=1002)			838 (83.6)
Caregivers (n=1002)			164 (16.3)

^a^The most frequently reported languages other than English were Hindi, Mandarin, Italian, Greek, and Arabic.

### Participant Enrollment

Overall, 17,820 part 1 booklets were distributed to hospitals to be provided to patients and caregivers, and participants had to complete a web-based enrollment form to receive subsequent components of the program. The conversion rate from booklets provided to actual enrollments was low, as only 1187 part 2 booklets were distributed to the enrolled participants. Most enrollments occurred following a hospital admission (733/1004, 73%), and 26.9% (275/1004) self-registered via the NHFA website.

Participants who had successfully completed the web-based registration form reported that web-based registration was simple. Health professionals and helpline staff reported barriers to onboarding, including confidence with technology, web-based form length, unclear information of the registration process, and absence of an email address (particularly for Aboriginal or Torres Strait Islander peoples). Health care staff, participants, and helpline staff reported that enablers may include (1) simplification and shortening of the web-based form, (2) providing feedback or monthly report to health professionals about enrolled patients to help maintain the program’s profile, (3) bedside enrollment by health professionals, and (4) raising awareness among health professionals, patients, and caregivers. The majority (34/38, 90%) of the health professionals reported that it was realistic to promote this program. The encouragement by health professionals was important to participants and was a key driver for the enrollment noted during participant interviews.

### Booklets

**Figure 4:**
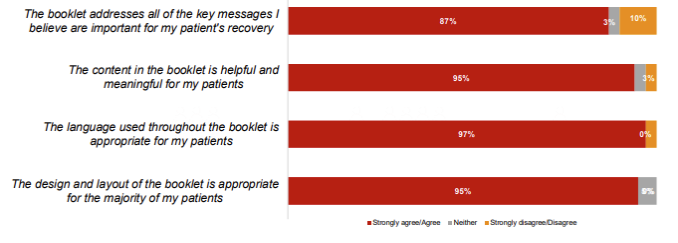
Responses to the question, "Below is a list of statements about the in-hospital booklet quick guide to heart attack and angina. Please indicate how strongly you agree or disagree with each statement.” (n=38 health professionals).

Overall, 94% (58/62) of the participants reported a high level of satisfaction with part 1 booklets and 89% (55/62) of them were satisfied with part 2 booklets. Most health professionals (35/38, 92%) reported that the part 1 booklet assisted with patient education and agreed that it addressed key messages, provided meaningful and helpful content, had appropriate language, and was well-designed (Figure 4). The hard copy version allowed professionals to fill information during the hospital journey at a time when patients absorb less information (Multimedia Appendix 1). Participants valued the hard copy format, diagrams, record-keeping tools, risk factor information, simple language, volume, and appropriate coverage of topics. One participant reported that “they’re informative about what to expect and if anything’s about to happen it gives you a little run-down on symptoms…I had no idea of any of that before.” The booklet helped the helpline staff who emphasized key information, for example, warning signs. Factors to be strengthened include the title, which was deemed less engaging than other NHFA resources, and minor changes to information about medications, triglycerides/cholesterol levels, alcohol consumption and driving, and stents.

### Text Messaging

**Figure 5:**
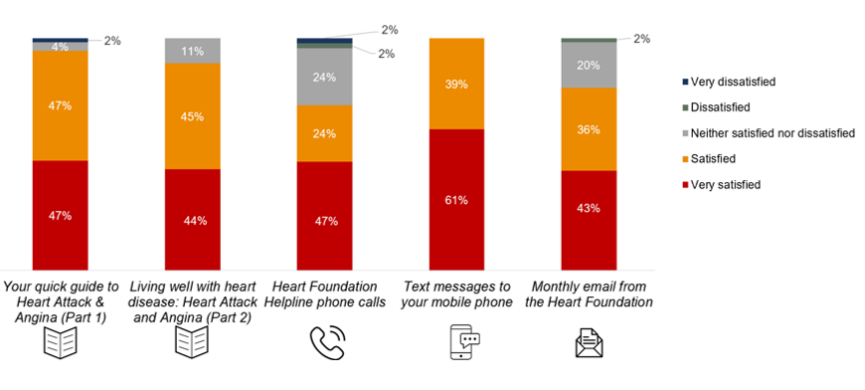
Response to the question, "How satisfied are you with the following elements of the Australian National Heart Foundation’s My Heart, My Life program?" (by participants at 3 months) (n=62).

Approximately 73% (733/1004) of the participants commenced the text messaging service, with 9% (90/1004) opting out. Reasons for opting out included feeling that messages were repetitive and were common sense. The majority (667/733, 90.9%) continued receiving messages until the end of the pilot period. Of those who responded to the survey, 88% (55/62) used the text messaging service and reported a very high level of satisfaction (Figure 5). The strengths of the text messaging service included emphasis on self-management through reminders, “And I keep getting tips about eating fruit and walking upstairs instead of taking elevators,” and “The little pointers are sort of helpful reminders that you need to keep exercising.” Minor suggestions included reducing repetitiveness across messages and providing links to further information, given that the texts were often succinct.

### Helpline

**Figure 6:**
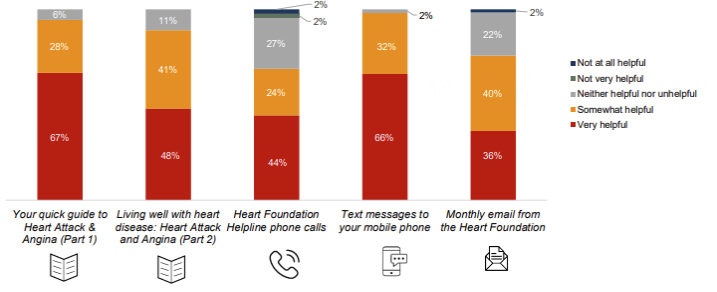
Response to the question, "In your opinion, how helpful have you found the following elements of the Australian National Heart Foundation’s My Heart, My Life program?" (by participants at 3 months) (n=62).

Outbound calls were provided to 978 participants, with 1691 calls made. Half of the participants who partook in the 3-month survey reported using this service, with 68% (42/62) reporting that it was helpful (Figure 6). The average call duration was 26 minutes (based on 1325 calls), with the first phone call taking 20 minutes or more. The helpline was unique in that it was individually tailored to participant circumstances, and participants liked that the helpline staff initiated the phone call. Suggestions for improvement included tailoring the frequency and duration of calls according to individual needs and removing the time limit.

### Emails

The data of email opening rates and clicking on content showed that there was good engagement of 964 participants with the email journey. Most continued on the journey, with only 11 participants opting out. Engagement rates in the first 2 emails were higher than those in subsequent emails. The MHML open rate (802/1129, 70.9%) and click-to-open rate (644/1129, 57.3%) (Figures S2 and S3 in Multimedia Appendix 4) were comparable to health care industry standards (24.3% and 11.2%, respectively) [9]. Participant interviews revealed that emails were helpful, as they provided useful information and helpful reminders, including the benefits of healthy eating patterns and recipes.

### Program Acceptability

At the 3-month survey, both participants and health professionals reported high levels of satisfaction with MHML (55/62, 88.7% and 33/38, 87%, respectively). All of them (62/62) reported being satisfied with the text message journey: 94% (58/62) and 89% (55/62) were satisfied with booklets 1 and 2, respectively; 79% (49/62) were satisfied with the monthly email journey; and 71% (44/62) were satisfied with the helpline calls. (Figure 4). All interviewed participants reported that they would definitely recommend the MHML program to others, as it provided a support for their recovery journey with information and resources complimenting cardiac rehabilitation, medical consultations, and health coaching. Suggestions from participants included adding webinars or forums delivered by health professionals, a peer support program for motivation and accountability, as well as a smartphone app with focus on healthy eating. Text messages and booklets were rated as the most helpful, followed by emails and calls (Figure 5). Health professionals supported continuation and expansion of the program. Improvements suggested by health professionals included expanding resources, especially for Aboriginal or Torres Strait Islander patients and cultural and linguistically diverse patients, including more hospitals, expanding to other cardiac conditions, and ensuring adequate staffing for rollout.

### Progress Toward Short-Term Outcomes

**Figure 7:**
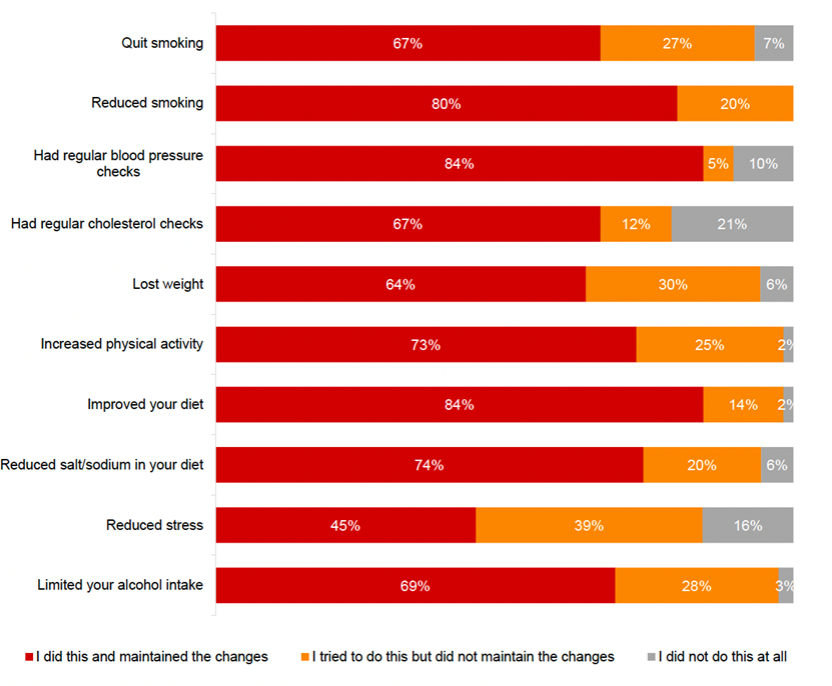
Participant lifestyle changes after the My Heart, My Life intervention program.

The self-assessed knowledge and confidence of the participants to manage their heart conditions were gauged at baseline, at the 3-month survey, and during interviews. Many participants reported trying to implement lifestyle behavior changes after joining the MHML, and a large proportion reported maintaining these changes after the program, for example, 73% (45/62) maintained increased physical activity and 84% (52/62) maintained a healthy diet (Figure 7).

## Discussion

### CHD Secondary Prevention Programs

MHML is a unique health care program delivered by the NHFA. Other organizations, including the British Heart Foundation and American Heart Association, promote telephone hotlines, web-based forums, and patient information pamphlets; however, a national, individualized, and multimodal program for CHD secondary prevention is yet to be implemented [10,11]. Economic modelling has shown that increasing referrals by 65% to secondary prevention support services such as cardiac rehabilitation could result in a saving of US $86.7 million over 10 years in health care costs [12]. Secondary prevention programs for cardiovascular diseases delivered by text messages have demonstrated to be potentially cost-effective [6,13]. The MHML program provides an additional comprehensive tailored support option without charge to those with CHD and their caregivers.

### Principal Findings

The pilot MHML program was well-received by participants and health professionals, the majority of whom reported that the program was easy to use (55/62, 88.7% of the participants and 33/38, 87% of the health professionals). Of note, much of the implementation of this pilot program was during the COVID-19 pandemic, thereby demonstrating the utility of alternative methods of support to patients with CHD at an otherwise vulnerable time point. The at-scale delivery of secondary prevention programs is challenging, and despite the knowledge that these programs are effective, little improvement has been made across the years in achieving comprehensive engagement of survivors of ACS in secondary prevention programs. The strengths of this program include the multimodal delivery of cardiac information by a centralized and respected organization, thereby supplementing existing care while encouraging self-management in secondary prevention.

Self-management strategies often lead to sustained behavioral change, and supporting patients on this journey to empower them is crucial [14]. Multiple behavior change techniques are more effective than a single technique with differences in individual preference and response [14]. By receiving a comprehensive and individualized program with physical booklets, program-initiated telephone support calls, text messages, and an email journey, participants in the MHML program were able to utilize multiple forms of communication to aid behavior changes. The uptake of this program was equivalently distributed across genders relative to the burden of CHD in these populations, wherein secondary prevention is underprescribed and underutilized in women, who have poorer cardiovascular outcomes [15]. This program appeared more accessible to a younger age group, and although many of lower socioeconomic status and some people of Aboriginal or Torres Strait Islander background accessed this program, it is important to optimize access for these population groups who historically have had poorer access to secondary prevention programs.

With regard to the feasibility of this program, there was overall satisfaction and acceptability in the feedback from health care professionals. The benefits of the MHML program were clearly expressed with the primary concern of implementation related to staffing of the service.

### Comparison to Other Studies

The participants found most aspects of the MHML program acceptable and helpful. Of note, they particularly valued the text messaging program, which has previously shown to alter cardiovascular outcomes in randomized controlled studies [6]. Given the relative ease of implementation at a pilot level, the NHFA MHML program should continue to expand to intensify the benefits of these complementary secondary prevention methods. Additional barriers such as COVID-19 were identified, and these barriers will continue to require innovative strategies to overcome the challenges. Alternative ways to reach patients and caregivers will need to be explored to allow widespread implementation of this program.

### Future Directions

Given the high levels of satisfaction regarding this pilot program, future directions should include a broader rollout of the MHML program. In particular, additional tools should be provided to support patient recovery (eg, webinars, smartphone apps), specific resources should be provided for patients of Aboriginal and Torres Strait Islander descent in addition to those from culturally and linguistically diverse backgrounds, and the program should be expanded to other hospital locations and include other types of cardiac conditions such as heart failure.

### Limitations

We acknowledge that the participants of the evaluation may have been more engaged with the program than those who had not engaged with the program. The invitation to participate in the surveys was distributed by email; therefore, participants who had responded may have been more skilled with technology than those who had not responded.

### Conclusions

The NHFA MHML pilot program was widely accepted by participants and health professionals. The NHFA is dedicated to improving recovery after a cardiac event and aims to improve and expand MHML by using evaluation findings. Future evaluations will consider more quantitative and clinical outcomes such as medication adherence and risk factor management to have an impact on the rates of unplanned rehospitalizations and the quality of life of participants.

## Appendix

Multimedia Appendix 1 Examples of the different components of the My Heart, My Life program.

Multimedia Appendix 2 List of hospitals participating in the My Heart, My Life pilot program.

Multimedia Appendix 3 Summary of data of participants of the My Heart, My Life program collected by the Australian National Heart Foundation.

Multimedia Appendix 4 My Heart, My Life program open rate and click-to-open rate data.

## Abbreviations

ACS: acute coronary syndrome
CHD: coronary heart disease
MACE: major adverse cardiac event
MHML: My Heart, My Life
NHFA: National Heart Foundation of Australia
TEXT ME: tobacco exercise and diet messages

## Data Availability

The data sets generated and analyzed during this study are available from the corresponding author on reasonable request.
