# Supplementary material for: Initial Implementation of the My Heart, My Life Program by the National Heart Foundation of Australia: Pilot Mixed Methods Evaluation Study
Source: JMIR Cardio. 2023 Oct 5;7:e43889. doi: 10.2196/43889 (PMC10587802; doi:10.2196/43889)
Supplement: Multimedia Appendix 1 [file cardio_v7i1e43889_app1.docx]

**Multimedia Appendix 1**. Examples of the MHML program

Figure S1: excerpts from the part 1 booklet demonstrating diagrams/pictures

| 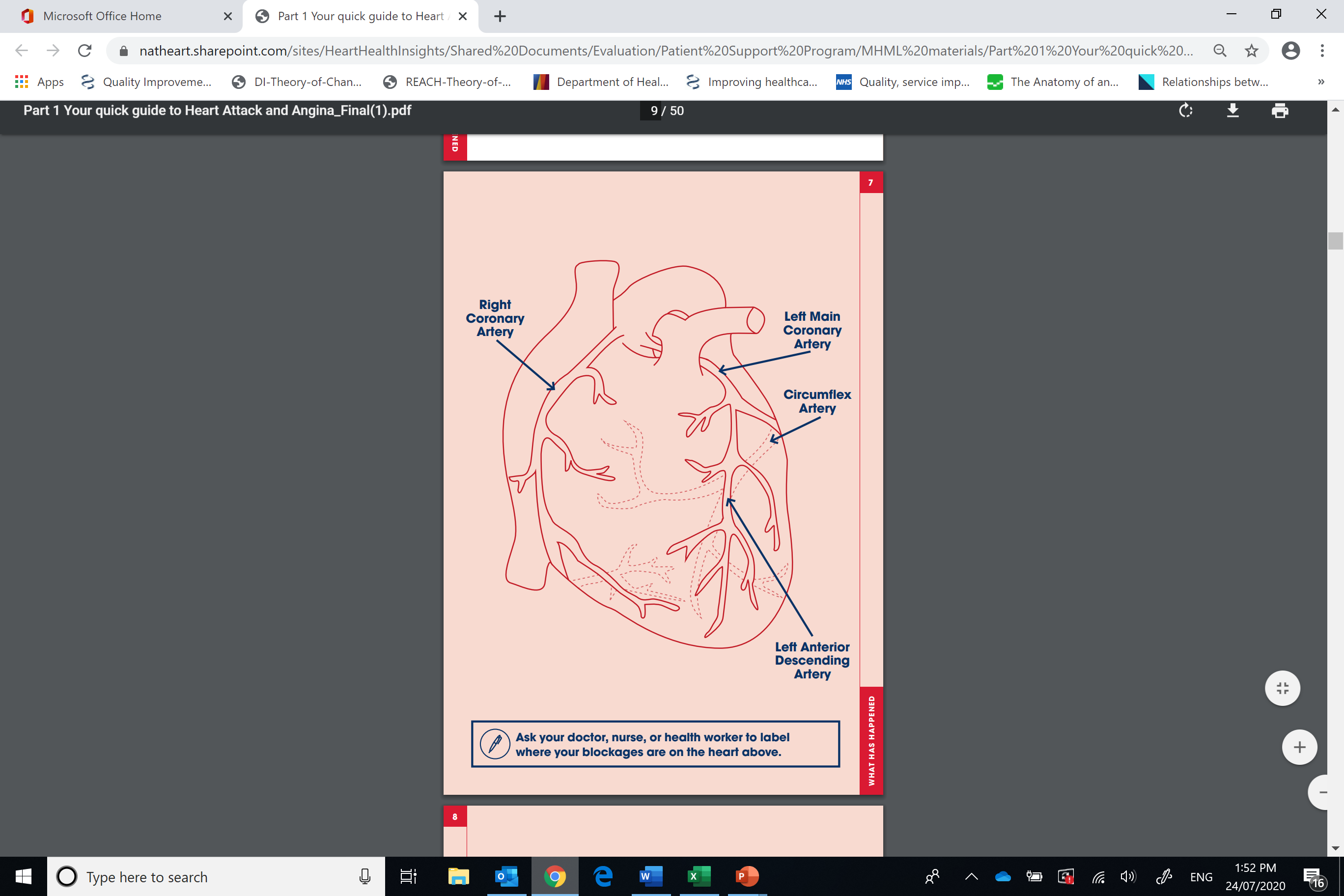 | 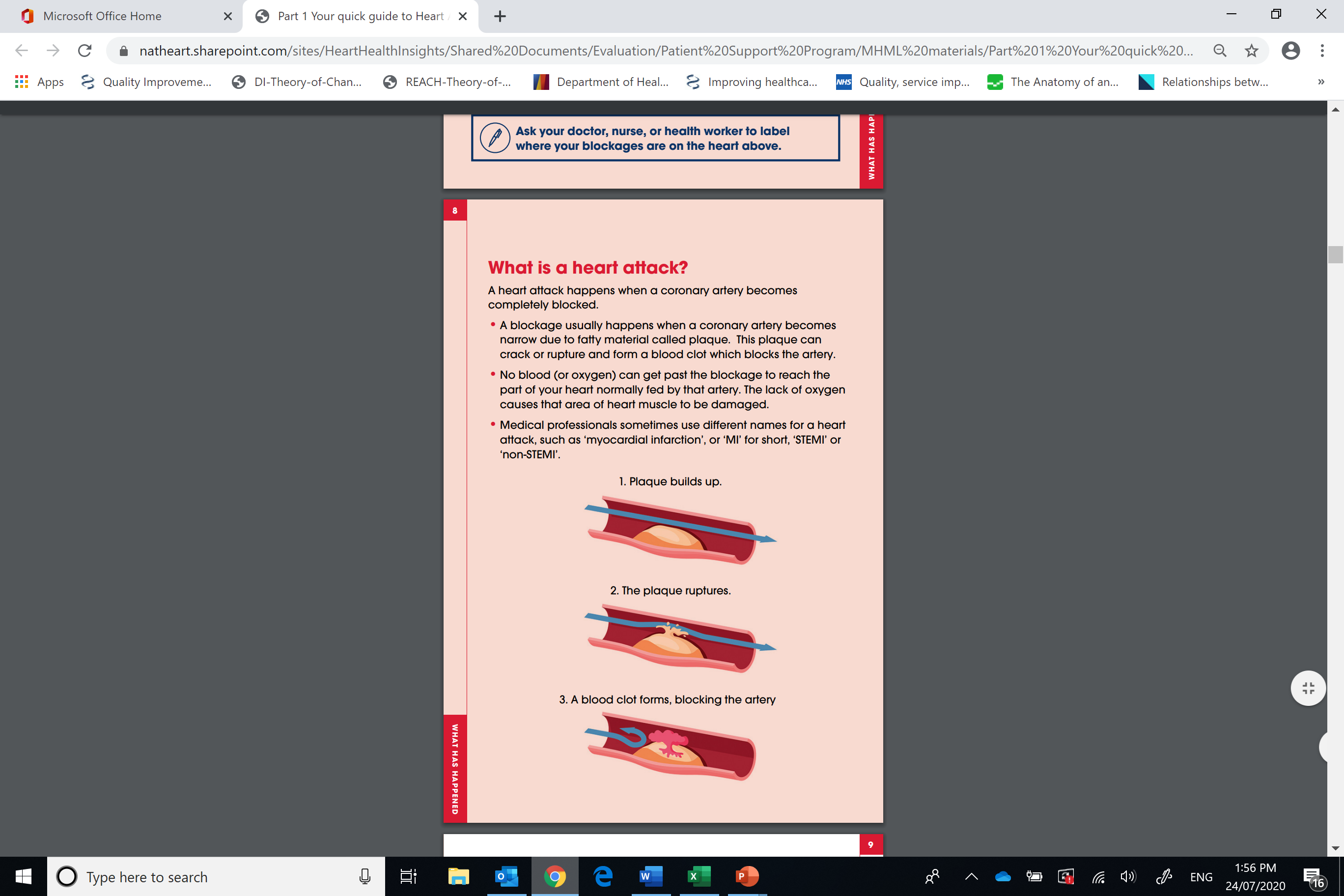 |
| --- | --- |
| Examples of text messages sent to pilot program participants   \| 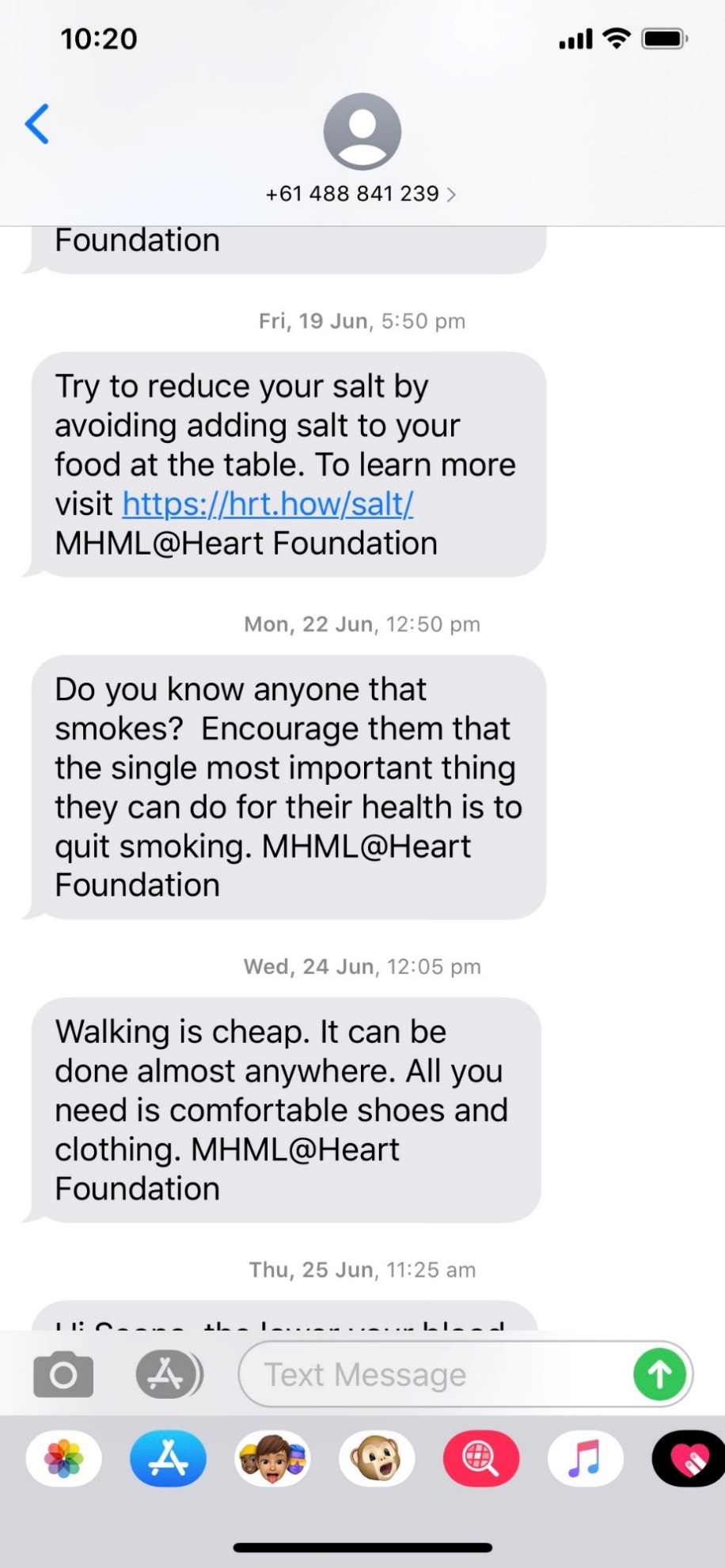 \|  \| \| --- \| --- \| | 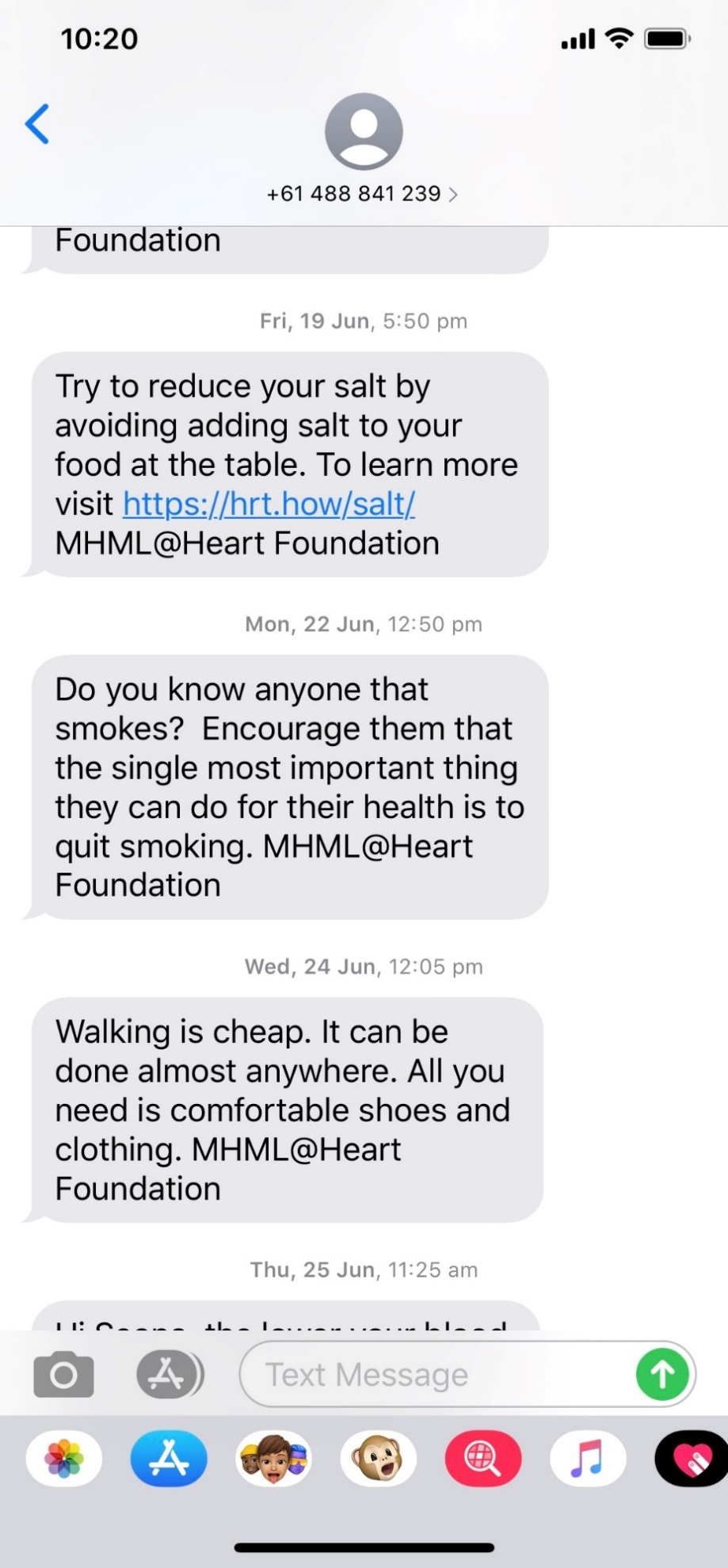 |
