# Supplementary material for: Initial Implementation of the My Heart, My Life Program by the National Heart Foundation of Australia: Pilot Mixed Methods Evaluation Study
Source: JMIR Cardio. 2023 Oct 5;7:e43889. doi: 10.2196/43889 (PMC10587802; doi:10.2196/43889)
Supplement: Multimedia Appendix 2 [file cardio_v7i1e43889_app2.docx]

**Multimedia Appendix 2. List of hospitals participating in the My Heart, My Life pilot program**

| **Hospital name** | **State or Territory** |
| --- | --- |
| Calvary Lenah Valley Hospital | ACT |
| Queanbeyan Community Health | ACT |
| Calvary Public Hospital | ACT |
| National Capital Private Hospital | ACT |
| The Canberra Hospital | ACT |
| Nepean Hospital | NSW |
| Royal Prince Alfred Hospital | NSW |
| Illawarra Heart Health Centre | NSW |
| Royal Darwin Hospital | NT |
| Alice Springs Hospital | NT |
| Darwin Private Hospital | NT |
| Central West Hospital Health Service | QLD |
| The Wesley Hospital | QLD |
| Central Queensland Hospital & H S | QLD |
| Caboolture Hospital | QLD |
| Princess Alexandra Hospital | QLD |
| Northwest Regional Hospital | TAS |
| Calvary St Vincent's Hospital | TAS |
| Hobart Private Hospital | TAS |
| Launceston General Hospital | TAS |
| Mersey Community Hospital | TAS |
| Royal Hobart Hospital | TAS |
| Bendigo Base Hospital | VIC |
| St Vincent's Hospital Melbourne | VIC |
| Box Hill Hospital | VIC |
| St John of God – Bendigo | VIC |
| Northern Hospital | VIC |
| Ballarat Health Service | VIC |
| Mount Hospital | WA |
| Armadale Health Service | WA |
| Sir Charles Gairdner Hospital | WA |
| Fiona Stanley Hospital | WA |
| St John of God Bunbury | WA |
| St John of God Subiaco Hospital | WA |
| Hollywood Private Hospital | WA |
| St John of God Hospital - Murdoch | WA |
| Royal Perth Hospital | WA |
| Joondalup Health Campus | WA |
