# Supplementary material for: Initial Implementation of the My Heart, My Life Program by the National Heart Foundation of Australia: Pilot Mixed Methods Evaluation Study
Source: JMIR Cardio. 2023 Oct 5;7:e43889. doi: 10.2196/43889 (PMC10587802; doi:10.2196/43889)

**Multimedia Appendix 3**. Summary of data collected by NHF for participants of MHML program collected by the Australian National Heart Foundation.

Table S1: Summary of data collected by NHF for participants of MHML program

| **Variable** | **Survey question** | **Scale** | **Timepoint** | | | |
| --- | --- | --- | --- | --- | --- | --- |
|  |  |  | **Baseline** | **3M** | **6M** | **12M** |
| Demographics | Age, Sex, Postcode | Multiple | ✓ |  |  |  |
| Self-management / Knowledge | How confident are you in…? | Very confident, Confident, Not very confident, Not at all confident, Unsure | ✓ | ✓ | ✓ | ✓ |
| Self-management / Knowledge | Reducing your risk of having a repeat heart event |  | ✓ | ✓ | ✓ | ✓ |
| Self-management / Knowledge | Being able to manage your heart condition |  | ✓ | ✓ | ✓ | ✓ |
| Self-management / Knowledge | Making lifestyle changes to help your heart condition (e.g. increasing physical activity, improving your diet, losing weight) |  | ✓ | ✓ | ✓ | ✓ |
| Healthcare utilisation | Have you been referred to cardiac rehab? | Yes, No, Prefer not to say | ✓ |  |  |  |
| Quality of Life | Would you say that in general your health is...? | E✓cellent, Very good, Good, Fair, Poor, Prefer not to say | ✓ | ✓ |  | ✓ |
| Quality of Life | Now thinking about your physical health, which includes physical illness and injury, for how many days during the past 30 days was your physical health not good? | Number of days, None, Unsure, Prefer not to say | ✓ | ✓ |  | ✓ |
| Quality of Life | Now thinking about your mental health, which includes stress, depression, and problems with emotions, for how many days during the past 30 days was your mental health not good? | Number of days, None, Unsure, Prefer not to say | ✓ | ✓ |  | ✓ |
| Quality of Life | During the past 30 days, for about how many days did poor physical or mental health keep you from doing your usual activities, such as self-care, work, or recreation? | Number of days, None, Unsure, Prefer not to say | ✓ | ✓ |  | ✓ |
| Self-management / Knowledge | How often do you have problems completing medical forms because of difficulty understanding the instructions...? | Always, Often, Sometimes, Occasionally, Never | ✓ | ✓ |  |  |
| Self-management / Knowledge | How often do you have problems learning about your medical condition because of difficulty understanding written information? | Always, Often, Sometimes, Occasionally, Never | ✓ | ✓ |  |  |
| Self-management / Knowledge | How confident are you filling out medical forms by yourself? | E✓tremely, Quite a bit, Somewhat, A little bit, Not at all | ✓ | ✓ |  |  |
| Organisational knowledge / confidence | Below are some statements that people have made about the Heart Foundation. Please indicate how: | Strongly agree, Agree, Neither agree nor disagree, Disagree, Strongly disagree, Unsure | ✓ | ✓ | ✓ | ✓ |
| Organisational knowledge / confidence | An organisation that I trust |  | ✓ | ✓ | ✓ | ✓ |
| Organisational knowledge / confidence | A respected organisation |  | ✓ | ✓ | ✓ | ✓ |
| Organisational knowledge / confidence | A leader amongst charities in Australia |  | ✓ | ✓ | ✓ | ✓ |
| Organisational knowledge / confidence | An organisation that's worthy of my support |  | ✓ | ✓ | ✓ | ✓ |
| Organisational knowledge / confidence | An organisation that makes a valuable contribution to the health of the Australian community |  | ✓ | ✓ | ✓ | ✓ |
| Organisational knowledge / confidence | Personally relevant to me |  | ✓ | ✓ | ✓ | ✓ |
| Organisational knowledge / confidence | Personally relevant to members of my immediate family |  | ✓ | ✓ | ✓ | ✓ |
| Organisational knowledge / confidence | Only relevant to people who have had a heart attack or have heart disease |  | ✓ | ✓ | ✓ | ✓ |
| Organisational knowledge / confidence | What is the likelihood of you donating money to the Heart Foundation in the ne✓t 12 months? | Very likely, Likely, Not very likely, Not at all likely, Unsure |  |  | ✓ | ✓ |
| Demographics | Firstly, are you......? | Living with heart disease, Have a family member/friend with heart disease, Care for someone with heart disease | ✓ | ✓ | ✓ | ✓ |
| Lifestyle risk factors | Since joining the Heart Foundation's My Heart My Life program, what changes, if any, have you made: | I did this and maintained the changes, I tried to do this but not maintain the changes, I did not do this at all, Not applicable, Unsure |  | ✓ | ✓ | ✓ |
| Smoking | Quit smoking |  |  | ✓ | ✓ | ✓ |
| Smoking | Reduced smoking |  |  | ✓ | ✓ | ✓ |
| Blood pressure | Had regular blood pressure checks |  |  | ✓ | ✓ | ✓ |
| Cholesterol | Had regular cholesterol checks |  |  | ✓ | ✓ | ✓ |
| Weight management | Lost weight |  |  | ✓ | ✓ | ✓ |
| Physical activity | Increased physical activity |  |  | ✓ | ✓ | ✓ |
| Diet | Improved your diet (e.g., increasing fruit, vegetable, and fibre intake) |  |  | ✓ | ✓ | ✓ |
| Diet | Reduced salt/sodium in your diet |  |  | ✓ | ✓ | ✓ |
| Mental health | Reduced stress |  |  | ✓ | ✓ | ✓ |
| Alcohol | Limited your alcohol intake |  |  | ✓ | ✓ | ✓ |
| Medication adherence | Have you been prescribed to take medications for your heart...? | Yes, No, Prefer not to say | ✓ | ✓ | ✓ | ✓ |
| Medication adherence | Which of the following would most closely describe your medication taking patterns over the past month? | I always take all my tablets at the same time of day, I manage to take all my tablets - but not always at the same time each day, I sometimes do not take all of my tablets, knowingly or unknowingly, but never omit more than one dose at a time, I miss many tablets, knowingly or unknowingly, and over the past month, I missed my tablets for two or more days, I take hardly any of my heart medication tablets, Prefer not to say | ✓ |  | ✓ | ✓ |
| Healthcare utilisation | Have you attended any cardiac rehab sessions? | Yes, No, Prefer not to say |  | ✓ | ✓ |  |
| Healthcare utilisation | Have you completed cardiac rehab? | Yes, No, Prefer not to say |  |  | ✓ |  |
| Healthcare utilisation | What was the main reason you have not attended cardiac rehab? | Have not been referred to a program, yet to attend, but have been enrolled in a program, Distance to the rehab centre, Transportation problems, Other commitments, Time constraints, Other health issues, Cost, No need to do rehabilitation, Other, please specify, Unsure |  |  | ✓ |  |
| Program acceptability | Please indicate if you are aware/and or used this resource/tool: | I am aware and frequently use this resource/tool, I am aware and infrequently use this resource/tool, I am aware but have not used this resource/tool, I am not aware of this resource/tool |  | ✓ | ✓ | ✓ |
| Program acceptability | Your quick guide to Heart Attack & Angina (Part 1) |  |  | ✓ | ✓ | ✓ |
| Program acceptability | Living well with heart disease: Heart Attack and Angina (Part 2) |  |  | ✓ | ✓ | ✓ |
| Program acceptability | Heart Foundation website |  |  | ✓ | ✓ | ✓ |
| Program acceptability | Monthly email from the Heart Foundation |  |  | ✓ | ✓ | ✓ |
| Program acceptability | Heart Foundation Helpline phone calls |  |  | ✓ | ✓ | ✓ |
| Program acceptability | Te✓t messages to your mobile phone |  |  | ✓ | ✓ | ✓ |
| Program acceptability | How satisfied are you with this resource/tool: | Very satisfied, Satisfied, Neither satisfied nor dissatisfied, dissatisfied, Very dissatisfied, Did not use |  | ✓ | ✓ | ✓ |
| Program acceptability | Your quick guide to Heart Attack & Angina (Part 1) |  |  | ✓ | ✓ | ✓ |
| Program acceptability | Living well with heart disease: Heart Attack and Angina (Part 2) |  |  | ✓ | ✓ | ✓ |
| Program acceptability | Heart Foundation website |  |  | ✓ | ✓ | ✓ |
| Program acceptability | Monthly email from the Heart Foundation |  |  | ✓ | ✓ | ✓ |
| Program acceptability | Heart Foundation Helpline phone calls |  |  | ✓ | ✓ | ✓ |
| Program acceptability | Te✓t messages to your mobile phone |  |  | ✓ | ✓ | ✓ |
| Program utility | How helpful have you found this resource/tool: | Very helpful, Somewhat helpful, Neither helpful nor unhelpful, Not very helpful, Not at all helpful, Did not use |  | ✓ | ✓ | ✓ |
| Program utility | Your quick guide to Heart Attack & Angina (Part 1) |  |  | ✓ | ✓ | ✓ |
| Program utility | Living well with heart disease: Heart Attack and Angina (Part 2) |  |  | ✓ | ✓ | ✓ |
| Program utility | Heart Foundation website |  |  | ✓ | ✓ | ✓ |
| Program utility | Monthly email from the Heart Foundation |  |  | ✓ | ✓ | ✓ |
| Program utility | Heart Foundation Helpline phone calls |  |  | ✓ | ✓ | ✓ |
| Program utility | Te✓t messages to your mobile phone |  |  | ✓ | ✓ | ✓ |
| Program acceptability | Overall, how satisfied are you with the Heart Foundation's My Heart My Life program? | Very satisfied, Satisfied, Neither satisfied nor dissatisfied, dissatisfied, Very dissatisfied, Unsure |  | ✓ | ✓ | ✓ |


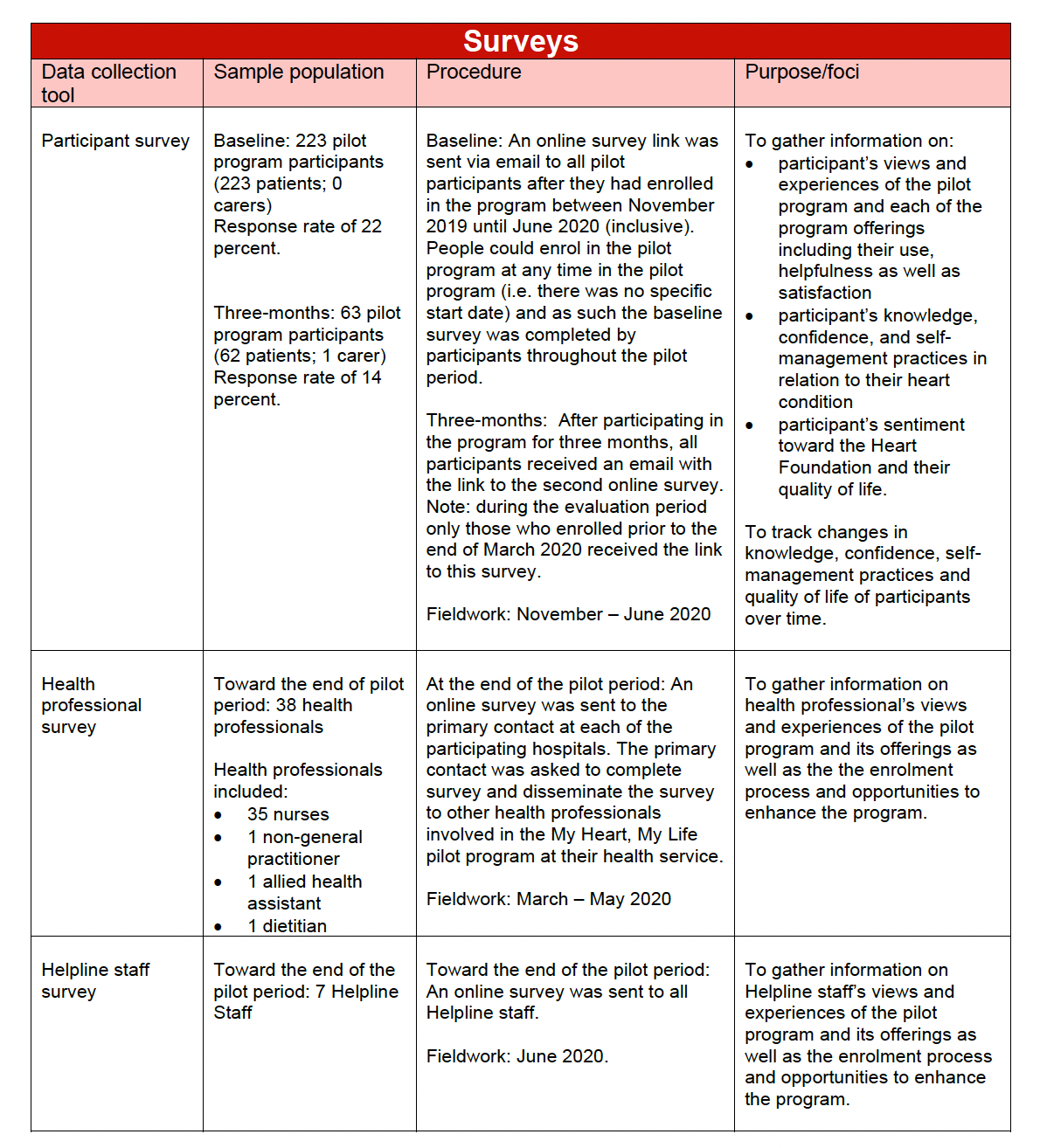
Table S2


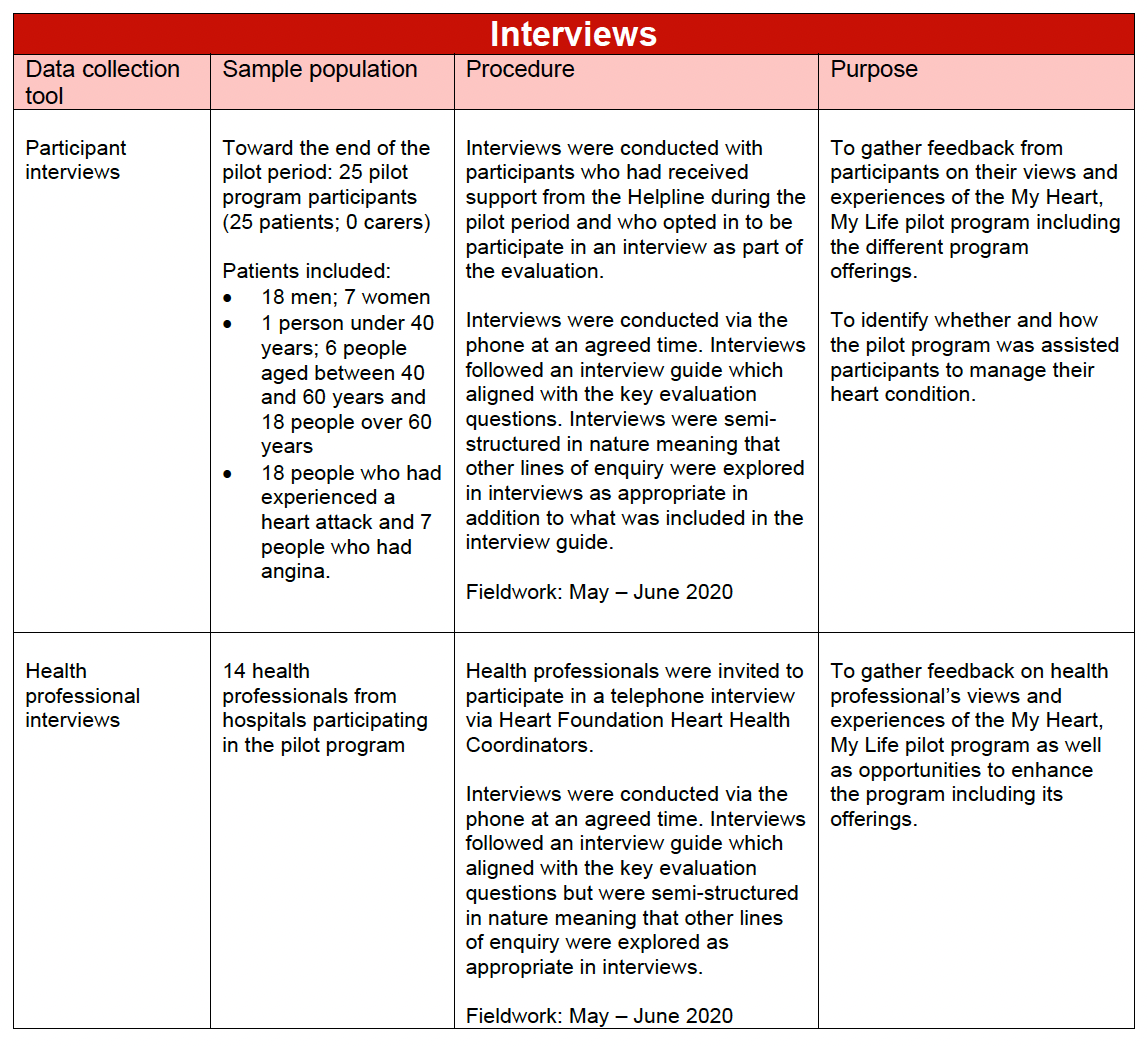

Supplement: Multimedia Appendix 3 [file cardio_v7i1e43889_app3.docx]
