# Supplementary material for: Initial Implementation of the My Heart, My Life Program by the National Heart Foundation of Australia: Pilot Mixed Methods Evaluation Study
Source: JMIR Cardio. 2023 Oct 5;7:e43889. doi: 10.2196/43889 (PMC10587802; doi:10.2196/43889)
Supplement: Multimedia Appendix 4 [file cardio_v7i1e43889_app4.docx]

**Multimedia Appendix 4**. MHML program open rate and click-to-open rate data.


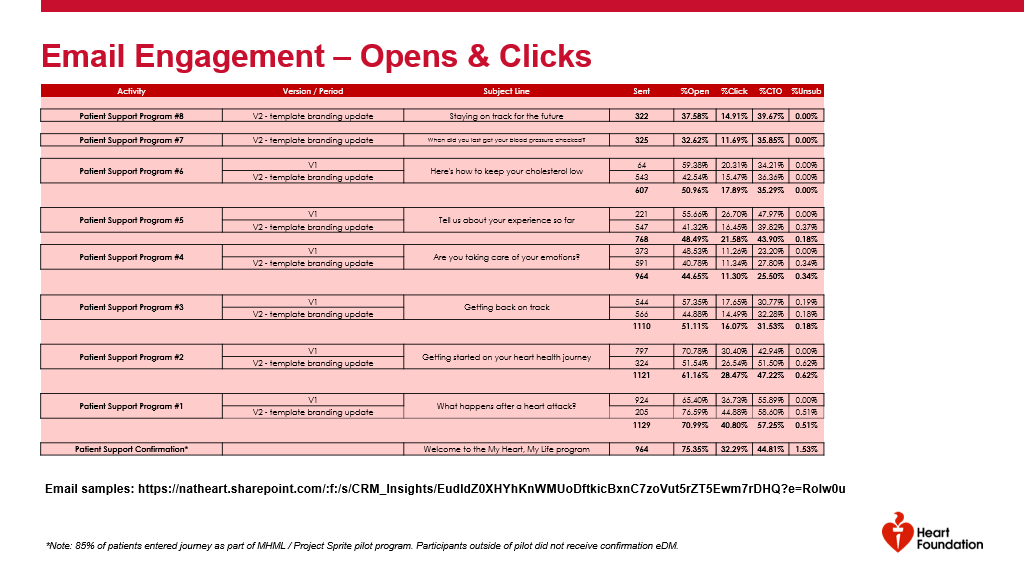


Figure S1. Gender of pilot program participants (patients) relative to the gender of patients hospitalised with CHD.


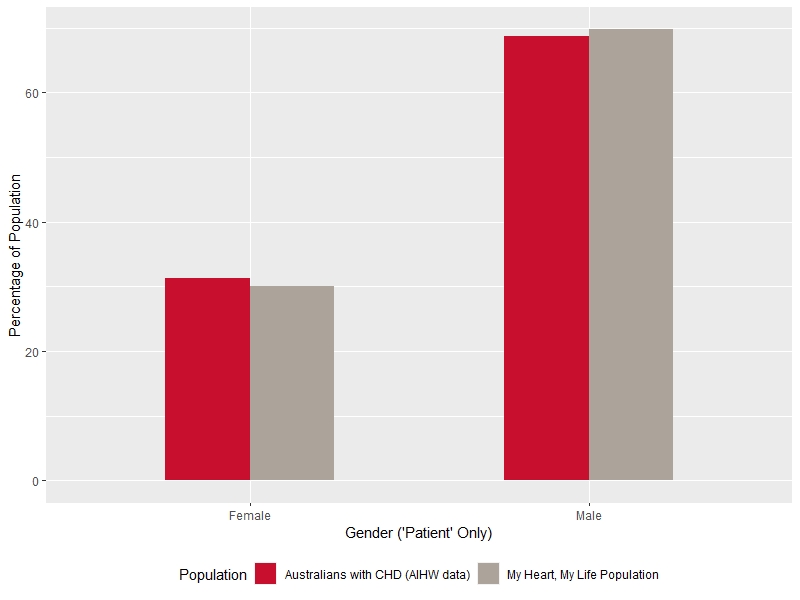


Figure S2. Proportion of people who received each of the emails and opened the message

**Figure S3**. Proportion of people who opened the email and clicked on links/contents. Demonstrates the click to open rate, a metric which compares the number of people who have opened an email with the number of people who have clicked on links/content contained in the message.
